# Influence of Acetylated Annealed Starch on the Release of β-Escin from the Anionic and Non-Ionic Hydrophilic Gels

**DOI:** 10.3390/pharmaceutics12010084

**Published:** 2020-01-20

**Authors:** Justyna Kobryń, Tomasz Zięba, Sandra Karolina Sowa, Witold Musiał

**Affiliations:** 1 Department of Physical Chemistry and Biophysics, Faculty of Pharmacy, Wroclaw Medical University, ul. Borowska 211A, 50-556 Wrocław, Poland; justyna.kobryn@umed.wroc.pl (J.K.); sandrusia360@wp.pl (S.K.S.); 2 Department of Food Storage and Technology, Faculty of Biotechnology and Food Science, Wroclaw University of Environmental and Life Sciences, ul. Chełmońskiego 37/41, 51-630 Wrocław, Poland; tomasz.zieba@upwr.edu.pl

**Keywords:** acetylated starch, β-escin, hydrogel, kinetics, Higuchi, Weibull

## Abstract

Naturally sourced products introduced to human nutrition and rediscovered for therapy include polysaccharides from potatoes. The starch may obtain unique properties via acetylation with acetic anhydride at 13 cm^3^/100 g of starch as the basic dose of reagent used in industrial conditions. The hydrogel formulation was applied as a carrier for escin included in the dry extract of *Aesculus hippocastanum*. Six hydrogels were evaluated (methylcellulose, polyacrylic acid-Carbopol 980 NF and polyacrylate crosspolymer 11—Aristoflex Velvet) with various concentrations of the modified starch. The kinetic studies of in vitro β-escin release were carried out in purified water at 37 ± 0.5 °C using a paddle apparatus at 50 rpm and a time period of 7 h. The criterion for the most suitable model was based on a high correlation coefficient of evaluated release profiles. The addition of modified annealed acetylated potato starch resulted in prolongation of β-escin release.

## 1. Introduction

Modern pharmaceutical technology is based, among others, on different carriers of medicinal substances, including synthetic and natural polymers. Among the latter, the high availability in plant sources should be considered an advantage, but the disadvantage is the variability of the composition, depending on the origin of the raw material. Starch and cellulose are known as very common natural polymers [1,2]. Previously used starch formulations, based on the pharmacopoeial rules, have been described as powders with different grain diameters. An alternative is semisynthetic polymers, e.g., modified cellulose or starch. Enzymatic, physical and chemical complex methods are used to modify starch molecules. The improvement of functional properties or the imparting of new, specific properties required for the desired application has been achieved. Modified starch (MS) is used, among others, in the food, textile, paper, construction and pharmaceutical industries. Adhesives, superabsorbents, biodegradable materials or heavy metal-absorbing beds are produced from MS [3]. Starch acetate is used in the food industry as a food additive. This modification is obtained on an industrial scale by esterifying starch in an aqueous suspension with acetic anhydride in an alkaline medium. It is characterized by a lower gelatinization temperature and higher viscosity of prepared pastes compared to those of natural starch and forms stable gels resistant to retrogradation.

An example of physical modification of starch is hydrothermal treatment, which includes annealing, i.e., native starch incubation with an excess of water at a temperature between the glass transition temperature and the initial gelatinization temperature. The purpose of this transformation is to receive such a modification temperature that stimulates the molecular mobility of starch molecules without transforming them to the gelatinized form. This process leads to an increase in the homogeneity and stability of the crystal structure of hardened starch, thereby protecting the structure of the final products [4,5]. The proposed technology allows for empty capsules to be prepared with interesting application features. Polymeric materials can affect the release kinetics of drug substances and provide controlled release of drugs in the appropriate area of the body. Therefore, research has been conducted on the effect of modified polymers of natural origin on the release kinetics of selected, model therapeutic substances [6,7].

Escin is the main active pharmaceutical ingredient in a horse chestnut seed (EH). It occurs as a mixture of triterpenoid saponins, existing in two forms, α-escin and β-escin (βE). They are characterized by different melting points, specific optical rotation, solubility in water and hemolytic index. βE is the major active component, and may be transformed into α-escin at elevated temperatures [8]. The application of EH in the form of hydrogel applied topically on the skin surface is an interesting alternative to peroral delivery of EH in the form of tablets used in the varicose veins [9].

The aim of the study was to evaluate the influence of the modified acetylated potato starch polymer (MSA) addition on the release profile of βE, depending on the amount of MSA added, and the type of basic hydrophilic gel, preformulated for the topical application.

A previous study on release kinetics from hydrogel formulations has been used, and the same cellulose and acryl derivatives polymers have been explored [9]. The previous study compared the release kinetics of βE from a variety of hydrogels prepared with commercially available polymers: Methylcellulose (MC), polyacrylic acid (Carbopol 980 NF, PA1), and polyacrylate crosspolymer 11 (PC-11), and from commercially available medicinal gel preparation with polyacrylic acid (Carbopol 5984). Formulations with MC presented the release profile comparable to those obtained from the marketed gel, whereas, the application of PA1, and PC-11 resulted in the prolonged release of βE [9]. Due to the present research, the addition of starch derivative—MSA—resulted in a stronger marked prolongation of the release.

## 2. Materials and Methods

### 2.1. Reagents

The following polymers were used as hydrogel components: Methylcellulose (MC 0512, Sigma Aldrich, Poznan, Poland), Carbopol 980 NF (PA1, Lubrizol, Wickliffe, OH, USA) and polyacrylate crosspolymer 11 (PC-11, Aristoflex Velvet, Clariant, Muttenz, Switzerland). The thick extract of *Aesculus hippocastanum* seeds (*Hippocastani seminis extractum spissum*, EH) from Herbapol S.A. (Wrocław, Poland) was used as a model medicinal substance. The modified acetylated potato starch polymer (MSA) from the University of Environmental and Life Science (Wrocław, Poland) was used at 20% and 40% as a studied component of the gels.

### 2.2. Hydrogel Preparation

The composition of the prepared gels is shown in Table 1. A concentration of 2.0% MC and 1.5% PC-11 and PA1 was prepared in gel formulations: F0, G0 and H0, respectively. The mixture with MC was achieved via adding distilled water of 80 °C and vigorously stirring in a mortar. The formulation with PA1 was completed by casting with 50.0 mg of 50% NaOH. The gels containing MSA were made with 20% and 40% of the starch derivative, respectively, by adding appropriate amounts of MSA instead of water: These were formulations F20 and F40, G20 and G40, as well as H20 and H40, respectively. The gels with MSA were gently mixed with a glass spatula to avoid foaming. The gels were conditioned in a refrigerator at 8 °C for 48 h. However, the temperature was brought to 25 °C before subsequent experiments.

### 2.3. Starch Preparation

**Figure 1:**
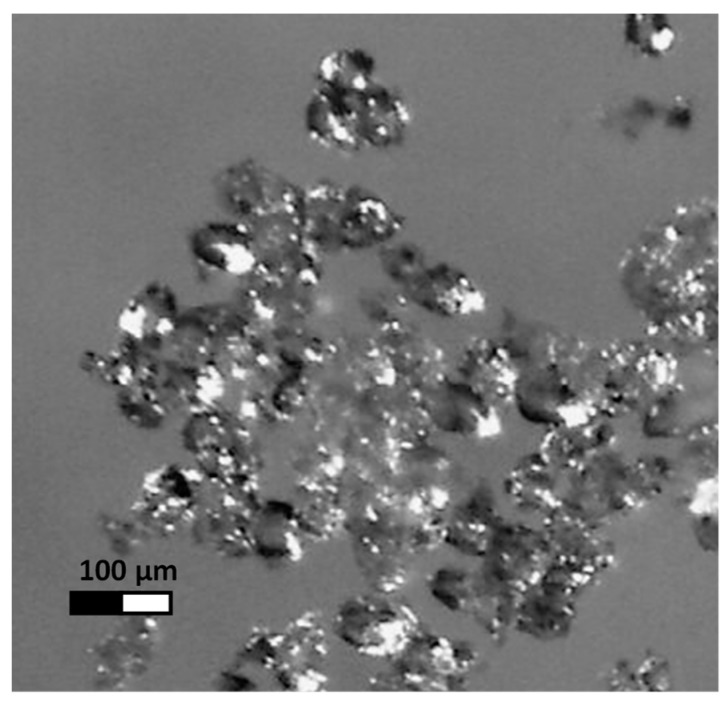
The optical microscopy visualization of the modified acetylated potato starch polymer (MSA).

A large starch knob fraction with an average volume diameter D(4,3) equal to 61.7 µm was isolated from natural potato starch with an average volume diameter D(4,3) of 39.1 µm, determined using a Malvern laser particle size analyser [4]. The large fraction of starch knobs was acetylated with acetic anhydride at 13 cm^3^/100 g of starch as the basic dose of reagent used in industrial conditions. After drying the acetylated starch, an initial gelatinization temperature of 49.17 °C using a Mettler differential scanning calorimeter was determined [5]. Two litres of a 10% starch suspension was prepared from acetylated starch and kept under constant stirring for 24 h at 48 °C. The starch was then washed three times with five-litre portions of distilled water. The starch pellet was separated from the suspension in each case using a Contifuge Stratos flow centrifuge (Heraeus, Germany) and then dried for 24 h in an air dryer at 30 °C. A photo of sample starch knobs was taken under a Stereo Zoom Microscope SMZ-171-TLED (Motic, Hong Kong, China) at fifty times magnification (Figure 1).

### 2.4. Kinetics Study

The release kinetics study was performed in a drug dissolution Tester Erweka GmbH DT 700 (Heusenstamm, Germany) paddle-over-disc apparatus using the pharmacopoeial method at a stirring rate maintained at 50 rpm. A dialysis tubing cellulose membrane 43 mm × 27 mm in size with pores of 14 kDa (Sigma-Aldrich, Saint Louis, MO, USA) was applied to the extraction cells with an area accessible to the dissolution of 28.26 cm^2^. Purified water was used as a dissolution medium in a volume of 900 mL at 37 ± 0.5 °C [10,11,12,13] without returning the medium. Six parallel measurements were performed in extraction cells by sampling 3 mL of the acceptor fluid every 5 min for 7 h. Analysis of the samples was performed by spectrophotometry using a UV/VIS spectrophotometer Jasco V-530 (Tokyo, Japan) at 265 nm, according to the available bibliography and comparing to the absorption spectrum of β-escin (βE) in an aqueous solution [14]. A standard curve based on three series of measurements with six concentration points from 59.0 to 236.0 μg/mL was generated. The results were examined according to zero-order kinetics, first-order kinetics, and second-order kinetics models [11,15,16], according to Higuchi [17]. The Weibull model was used as a preference statistic model for the hydrogel formulations, and Statistica software was used to predict the plot of the release kinetics [18]. 

The models applied in the study (Table 2) come from the Fick’s first law, which may be interpreted in the context of the diffusion process through the semi-permeable membrane. The general equation is presented as below (Equation (1)),

(1) $$J=-D\frac{dC}{dx},$$

where *J* is the amount of solute passing through a unit area perpendicular to the surface per unit time, *D* is the diffusion coefficient, and *dC/dx* is the concentration gradient, which represents a driving force for diffusion [19]. There are conditions which must be met to apply zero-order model: Dissolved drug form does not aggregate, dosage form surface does not change, and no equilibrium state is achieved. This type of model usually concerns the prolonged release process [17]. The first-order model shows the connection between the dissolution of solid particles and surface action [19,20]. In second-order kinetics model release, process depends on the time units and concentration both. Higuchi model is usually applied to the release of water-soluble and poorly water-soluble drugs included in semi-solid and/or solid matrices [19]. The Weibull model is frequently used in all kinds of dissolution curves [17].

## 3. Results

### 3.1. Kinetics

#### 3.1.1. Zero-Order Kinetics Model

**Figure 2:**
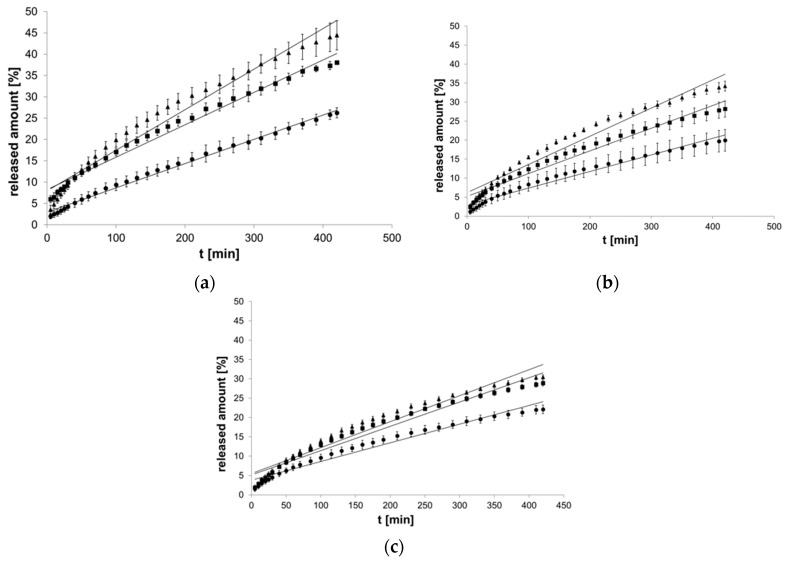
Process of release kinetics of β-escin (βE) performance according to zero-order kinetics model; (**a**) MC gel formulations, (**b**) PA1 gel formulations, (**c**) gel formulations with PC-11; ▲—reference gel, ■—20% of MSA, ●—40% of MSA, *n* = 6.

The initial amount of EH in the formulations is presented in Table 1. The plateau phase was established after 420 min. The largest released amount of βE in the equilibrium state was 44.43% and belonged to MC formulation (Figure 2a), and the smallest was 19.98% for PA1 hydrogel (Figure 2b). Generally, when more MSA was added to the formulation, less βE was released (Figure 2). In the case of the formulations with MC and PA1, 40% MSA addition caused the released amount to decrease by almost half (Figure 2a,b). In the hydrogels with PC-11, MSA supplementation exhibited an approximate 30% decrease (Figure 2c). The release processes did not follow the linear course of the zero-order kinetics function (Figure 2).

#### 3.1.2. First-Order Kinetics Model

**Figure 3:**
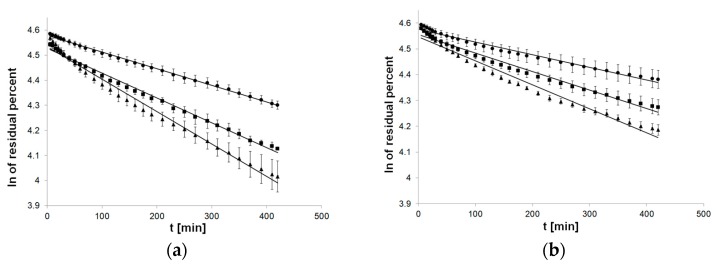

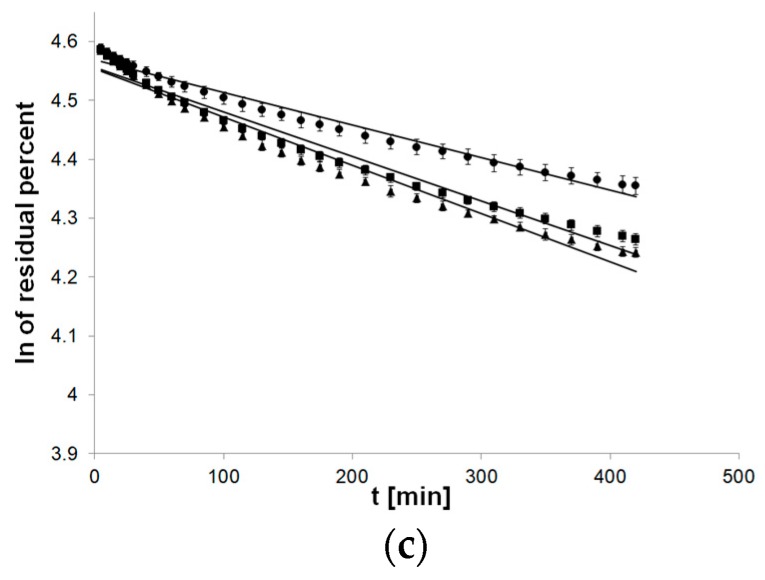
Process of release kinetics of βE performance according to first-order kinetics model; (**a**) MC gel formulations, (**b**) PA1 gel formulations, (**c**) gel formulations with PC-11; ▲—reference gel, ■—20% of MSA, ●—40% of MSA, *n* = 6.

The flow of first-order kinetics model function indicated a departure of linearity, especially applied to formulations with PC-11 (Figure 3).

#### 3.1.3. Second-Order Kinetics Model

**Figure 4:**
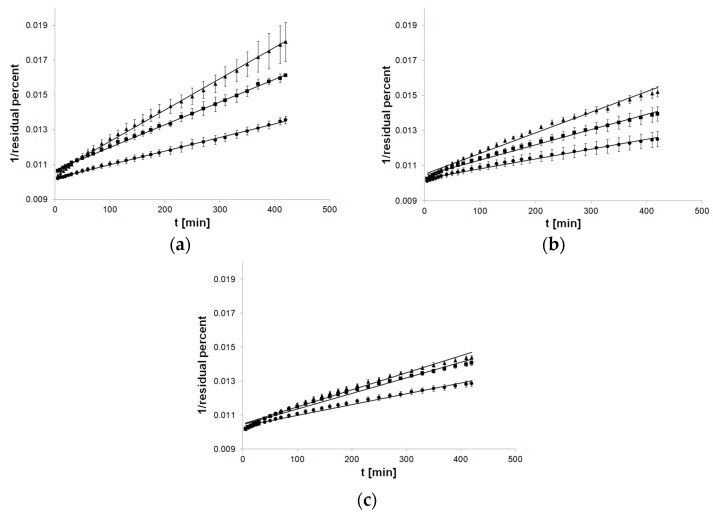
Process of release kinetics of βE performance according to second-order kinetics model; (**a**) MC gel formulations, (**b**) PA1 gel formulations, (**c**) gel formulations with PC-11; ▲—reference gel, ■—20% of MSA, ●—40% of MSA, *n* = 6.

The charts for the second-order kinetics model were well suited to the linear course of the function, especially used to formulations with MC (Figure 4).

#### 3.1.4. The Higuchi Model

**Figure 5:**
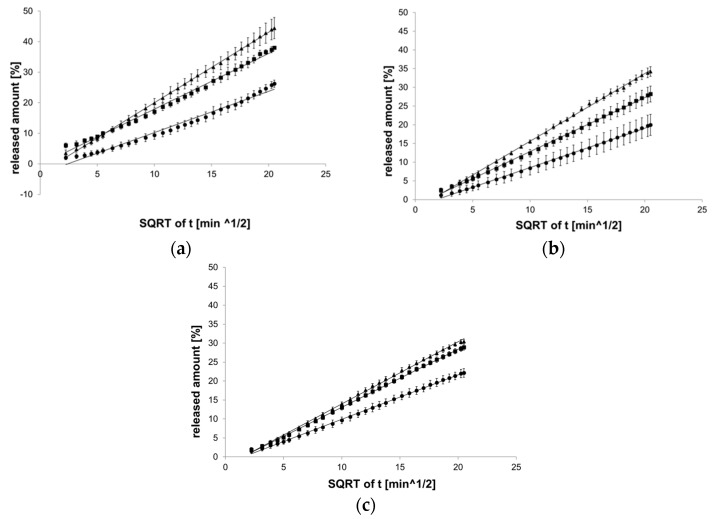
Process of release kinetics of βE performance according to the Higuchi model; (**a**) MC gel formulations, (**b**) PA1 gel formulations, (**c**) gel formulations with PC-11; ▲—reference gel, ■—20% of MSA, ●—40% of MSA, *n* = 6.

The Higuchi model best reflected the course of reactions with PC-11 hydrogel (Figure 5).

#### 3.1.5. The Weibull Model

**Figure 6:**
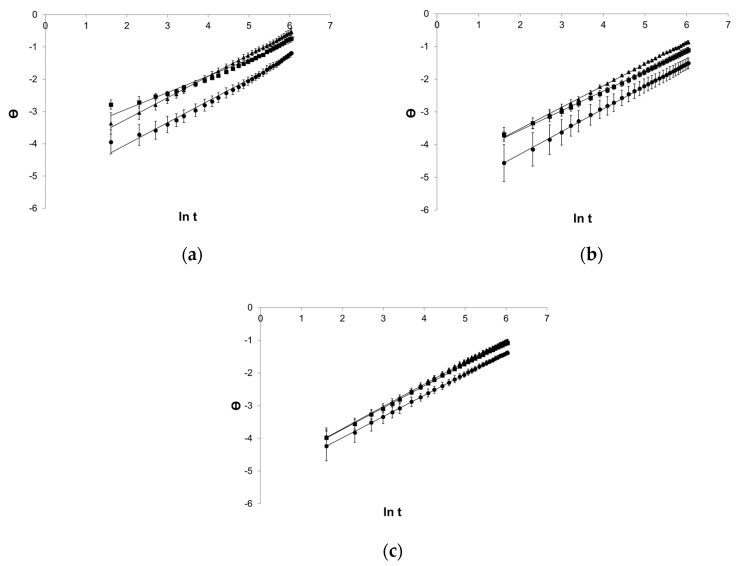
Process of release kinetics of βE performance according to the Weibull model; (**a**) MC gel formulations, (**b**) PA1 gel formulations, (**c**) gel formulations with PC-11, Θ—ln[−ln(1 − (released percent/100%))], ▲—reference gel, ■—20% of MSA, ●—40% of MSA, *n* = 6.

The logarithmic equation of the Weibull model introduced a good fit for the rectilinear course of graphs (Figure 6).

**Figure 7:**
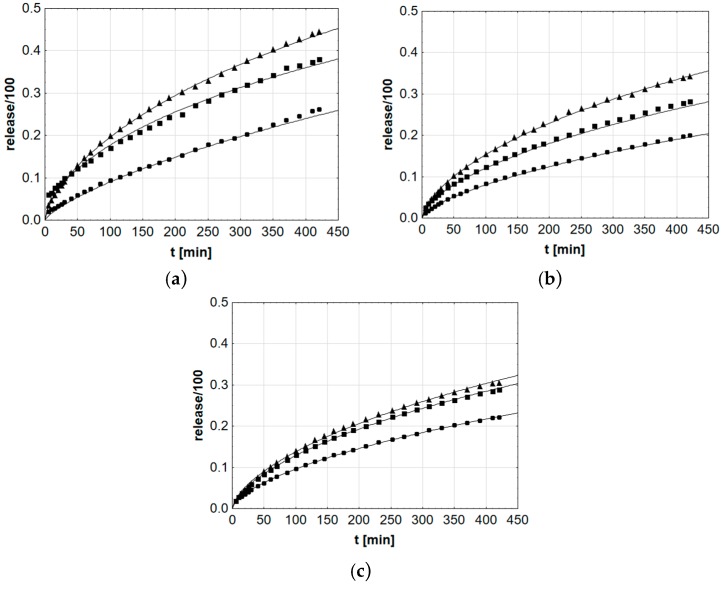
Courses of mean βE release in the Weibull model evaluated in Statistica application, applied to formulations (**a**) MC, (**b**) PA1, and (**c**) PC-11, ▲—reference gel, ■—20% of MSA, ●—40% of MSA.

Additionally, the probable course of release reactions in the Weibull model was statistically adjusted (Figure 7).

#### 3.1.6. Kinetics Models Parameters

The range of the release rate constants of the formulations was from 4.37 × 10^−2^ %·min^−1^ to 9.50 × 10^−2^ %·min^−1^ (Table 3). The best match belonged to the second-order kinetics and Higuchi model, as shown in Table 3. The release rate constants for the second-order kinetics were in the range of 5.59 × 10^−6^ %^−1^·min^−1^ to 1.80 × 10^−5^ %^−1^·min^−1^. The Higuchi model showed a minimum release rate constant of 1.05 %·min^−1/2^ and a maximum release rate of 2.31 %·min^−1/2^ (Table 3). The calculated values of the shape parameter β in the Weibull model were between 0.598 and 0.769 in preparations F20 and F40, respectively. The constants and regression coefficients are shown in Table 3. The range of the regression coefficients of the kinetics models was from 0.9522 to 0.9992, and the highest was observed for the H20 formulation. The Weibull model showed regression coefficients between 0.9889 and 0.9993 (Table 3).

**Figure 8:**
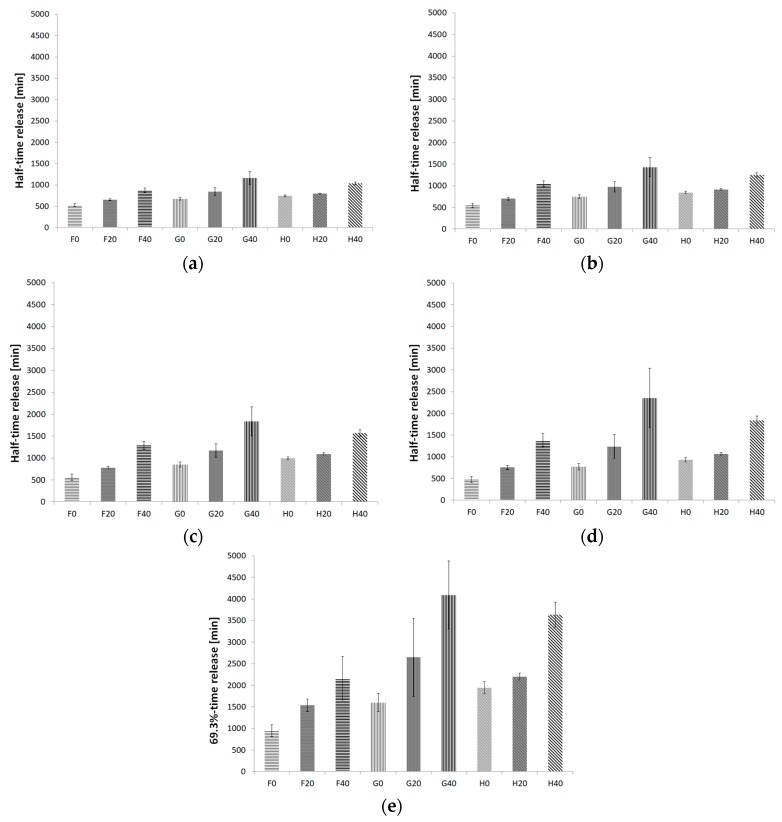
Half-time release and 69.3%-time release histograms of βE for the formulations with hydrogels of MC (F0, F20, F40), PA1 (G0, G20, G40) and PC-11 (H0, H20, H40), evaluated according to the kinetics models: (**a**) zero-order kinetic equation, (**b**) first-order kinetic equation, (**c**) second-order kinetic equation, (**d**) Higuchi model, (**e**) Weibull model; the error bars represent SD, *n* = 6.

Figure 8 shows the half-time release for the kinetics models and 69.3%-time release for the Weibull model for all formulations. The range of the half-time release in the zero-order model was 528.21 min to 1161.55 min. For first-order kinetics, the range of half-time release of βE was 539.29 min to 1432.08 min. For second-order kinetics, the minimal half-time release was 563.65 min, and the maximal was 1833.73 min. The Higuchi model showed a range of 475.72 min to 2354.88 min. The Weibull model showed the shortest time release of 69.3% βE after 949.26 min and the longest after 4091.24 min. The minimal values were exhibited for the F0 and the maximal for the G40 formulations according to the kinetics and Weibull models.

## 4. Discussion

The best kinetics model match was chosen for the studied hydrogel formulations according to the highest correlation coefficients, as presented in Table 3. The formulations with MC hydrogel showed the best match to the Higuchi model (F0) and the second-order kinetics model (F20, F40). The PA1 and PC-11 formulations released the active substance in accordance with the Higuchi model. The worst fitting kinetics were observed for the zero-order kinetics model considering all formulations. This suggests the course of release depended on substances concentration in the donor compartment. Moreover, in the case of first-order kinetics, we did not obtain the very high values of *r*^2^ coefficient. Due to the known theory, the first-order process kinetics depends on the concentration of one molecular species engaged in the release. However, there should be another additional factor, as the *r*^2^ coefficient are not very high. The evaluation of second-order process was motivated by our scientific interest and curiosity, if maybe another molecular species may influence the process parallelly with the active substance. Surprisingly we obtained very high values of r^2^ coefficient in selected cases. This leads us to the concept which should be further studied, that two dynamic factors influence the release.

Our previous study results were compared, and similar results have been observed [9]; additionally, the Higuchi model has been complemented. The Higuchi model is based on a few parameters. Many factors may influence the release rate process, including following assumptions: The substance concentration exceeds its solubility, the matrix thickness is much larger than the size of the active substance, the swelling and dissolution of the matrix has no effect on the process, and there is a one-way diffusion and “sink conditions” are preserved, and the substance already present in the solution does not have a significant effect on the rate of release of its remaining amount [16]. In the case of second-order kinetics, a change in the concentration of two substrates influences the process rate [10]. The observed decreased rate of the release process might have been impacted by the interaction of MSA with the components of the hydrogels, as well as the active substance itself. This was more likely in the PA1 and PC-11 hydrogels than in MC because of the presence of reactive functional groups. The methyl groups in MC are substituted with hydroxyl groups at positions C2, C3 and/or C6 on glucose. This molecule is non-ionic and has an amphiphilic character. The structure of the MC makes it flat and stiff [21]. The heterogeneous distribution of the methyl groups and degrees of substitution larger than 1.3 enables hydrophobic interactions of the polymer [22]. As mentioned in our previous study [9], the anionic character of polyacrylic acid (PA1) may facilitate the production of complexes with cationic residues of other compounds [23]. In the case of the PC-11 hydrogel, cationic properties were observed. The amide groups may bond to the carboxyl groups of βE on the basis of hydrogen bonding. Navarro et al. investigated interactions between the carboxyl group of benzoic acid and the amide group of cyclophane. They found superiority of the O_carboxyl_–H· ·O=C_amide_ bond over the C=O_carboxyl_· ·H–N_amide_ bond as a result of a large negative charge density on the C=O oxygen [24]. The MSA was formed by means of acetic anhydride esterification, according to the method described by Zieba et al. [25]. The reaction proceeds as follows (Equation (2)) [26]:

(2) $$starch-OH+CH_{3}COOCOCH_{3}+NaOH\to starch-OCOCH_{3}+CH_{3}COONa+H_{2}O$$

**Figure 9:**
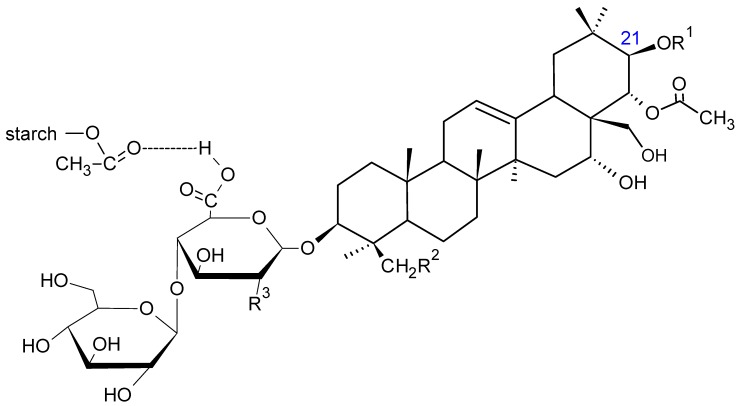
The scheme of possible hydrogen bonding between the carboxyl groups of MSA and βE [27].

Considering the aqueous conditions of the reaction, there is a likelihood of hydrogen bonds between the carbonyl groups of MSA and βE (Figure 9).

The most interesting parameter which reflects the expected release prolongation is the half-release time calculated on various equations according to available sources [9]. The release rate is inversely proportional to the half-release time. However, the half-release time units enable simple comparisons. The respective half-release times are presented in Figure 8. The release rates, as presented in Table 3, were between 4.37 × 10^−2^ %·min^−1^ and 9.50 × 10^−2^ %·min^−1^ for the zero-order kinetics and between 4.93 × 10^−4^·min^−1^ and 1.29 × 10^−3^·min^−1^ for the first-order kinetics. For the second-order kinetics, they were between 5.59 × 10^−6^ %^−1^·min^−1^ and 1.80 × 10^−5^ %^−1^·min^−1^, and for the Higuchi model, they were between 1.05 %·min^−1/2^ and 2.31 %·min^−1/2^. The highest values of release rate were observed in the case of the formulations with MC, and the lower values were similar to each other in the case of the preparations with PA1 and PC-11. MC was applied in the formulations F0, F20, and F40. MC exhibits water- and organo-solubility and thermal gelation properties. Its wide range of viscosity (5–75,000 mPa·s at 2%) corresponds to an average molecular weight range of 10,000–220,000 Da, making it useful as a carrier, a coating agent, and a disintegrant for tablets and matrix tablets [28]. MC 0512 used in our study had a viscosity of 4000 mPa·s, which is a medium-compact property, compared with that of the formulations with acrylic acid and modified acrylic acid (PA1 and PC-11), which show hydrophilic properties. The viscosity range of the PA1 hydrogel was approximately 60,000 mPa·s at 1.5%, and that of PC-11 was approximately 20,000 mPa·s at 1.5% [29]. Additionally, the viscosifying properties of polyacrylic acid polymers are strongly dependent on the molecular weight, degree of cross-linking, level of hydrophobic alkyl groups in their backbone, pH and concentration in the formulation. Additionally, the charge character of acrylate-based polymers is typically imparted by carboxylic acid functionality, and is, therefore, dependent upon the pH of the formulation. At pH above 3.5, the carboxyl groups exhibit a negative charge and interact with polar solvents, such as water, as it is in the case of presented preparations. Moreover, the polymer strands uncoil into a swollen structure owing to internal crosslinks [29]. The ability of chitosan hydrogel, whose structure includes amino groups, to engage in ionic crosslinking with other chemical compounds has been indicated by many scientists, such as Muzzarelli [30], Berger [31] and Mahdavinia [32]. The above-presented factors may modify the βE release in specific hydrogels; however, the MSA addition tends to prolong the βE release in all the cases—the formulations: F20, F40, G20, G40, H20, H40.

According to the Weibull model, a β-factor has been established (Table 3). The range of β was between 0.598 and 0.769, and the average value of β was 0.656. The lowest value was observed for F20, and the highest was observed in the case of F40. Several authors have treated the dependency of the β-value on the release mechanism of active substances. Papadopoulou et al. showed a dependence of the β-value up to 0.75 on the Fickian diffusion mechanism. In the β range between 0.75 and 1.0, Fickian diffusion is connected with case II transport. The presence of β-values greater than 1.0 highlights a complex release mechanism [33]. Peppas and coworkers [34] have interpreted a β-value of 0.5 as an indicator of Fickian law-dependent diffusion, where a β-value equal to 1.0, the drug release rate is independent of time (corresponding to zero-order release kinetics and case II transport). The values of β between 0.5 and 1.0 indicate the mixed mechanism of both (anomalous transport). Furthermore, Siepmann et al. [35] interpreted β = 0.5 as a diffusion-controlled drug release meter and β = 1.0 as an indicator of swelling-controlled drug release. Fickian law deviation is connected with the finite rate of the release reaction at which hydrophilic polymers may be relaxed to accommodate water [36]. Hopkinson et al. [37] studied the effect of water on amylose using stray field NMR. They demonstrated characteristics of case II transport for the ingress of liquid water. Similarly, Gümüşderelioğlu and Kesgin [38] tested the release of bovine serum albumin from poly(vinyl ether)-based hydrogels and observed the absorption of water and the release of a drug via a swelling-controlled diffusion mechanism that occurred simultaneously. The range of β was 0.46 and 0.84, depending on the pH of the loaded gel. They recognized the existence of some molecular relaxation processes in addition to diffusion as responsible for the Fickian deviation.

## 5. Conclusions

The influence of MSA concentration in the evaluated hydrogel formulations on the release rate of βE was confirmed. The formulations of MSA with MC (F40) and PA1 (G40) had the highest impact on the release prolongation. The highest release prolongation was obtained in the formulation G40 containing PA1 and MSA. The PC-11 (H0) itself decreased βE release, whereas, the MSA addition slightly influenced the process (H40). Studies are still needed on the mechanism of binding the active substance to the PC-11 hydrogel, as there may exist strong interaction of βE with the polymer, presumably via the amide bonds. This may be ascribed to the presence of amide groups bonding to the carboxyl groups of βE and carboxylic groups interacting with polar solvents, such as water. The best fit of βE release pattern belonged to second-order kinetics and the Higuchi models. The Weibull model adapted to the experimental data. The appearance of β-value between 0.5 and 1.0 pointed to the mixed mechanism of the occurred processes.

## Figures and Tables

**Table 1:** The composition of the formulations with *Hippocastani seminis extractum spissum* (EH): F—MC hydrogels, G—PA1 hydrogels, H—PC-11 hydrogels.

Formulation	Composition (g)					
	EH	MC	PA1	PC-11	MSA	H_2_O
F0 *	7.08	1.2	-	-	-	51.72
F20	7.08	1.2	-	-	12.0	39.72
F40	7.08	1.2	-	-	24.0	27.72
G0 *	7.08	-	0.9	-	-	52.02
G20	7.08	-	0.9	-	12.0	40.02
G40	7.08	-	0.9	-	24.0	28.02
H0 *	7.08	-	-	0.9	-	52.02
H20	7.08	-	-	0.9	12.0	40.02
H40	7.08	-	-	0.9	24.0	28.02

* Formulation of analogous composition was applied in [9].

**Table 2:** Kinetic models applied to estimate obtained results. *Q*_0_ is initial percentage of the released drug; *Q*_*t*_ is percentage of the released drug after time *t*; *K* is rate constant, for zero-order kinetics (*K*_0_), 1st-order kinetics (*K*_I_), 2nd-order kinetics (*K*_II_), and Higuchi model (*K*_*H*_), respectively; *Td* is time after release of 69.3% of drug from the formulation; *t*_0.5_ is half release time; *β* is a shape parameter in the Weibull model; *a*, *b*, are slope and intersection of the graph representing the release process, respectively.

Models Applied	General Equation	Parameters	
Zero-order	$Q_{t}=Q_{0}-K_{0}t$	$K_{0}=\frac{Q_{0}-Q_{t}}{t}$	$t_{0.5\left(0\right)}=\frac{Q_{0}}{2K_{0}}$
First-order	$Q_{t}=Q_{0}e^{-K_{I}t}$	$K_{I}=\frac{1}{t}ln\frac{Q_{0}}{Q_{t}}$	$t_{0.5\left(I\right)}=\frac{0.693}{K_{I}}$
Second-order	$\frac{1}{Q_{t}}=\frac{1}{Q_{0}}+K_{II}t$	$K_{II}=\frac{Q_{0}-Q_{t}}{Q_{0}Q_{t}}\frac{1}{t}$	$t_{0.5\left(II\right)}=\frac{1}{K_{II}Q_{0}}$
Higuchi	$Q_{t}=Q_{0}-K_{H\;}t^{0.5}$	$K_{H}=\frac{Q_{0}-Q_{t}}{t^{0.5}}$	$t_{0.5\left(H\right)}={\left(\frac{50}{K_{H}}\right)}^{2}$
Weibull	$Q_{t}=(100-Q_{0})$ $\left[1-e^{-{\left(\frac{t}{T_{d}}\right)}^{\beta }}\right]$	$\beta =log_{\left(-\frac{t}{T_{d}}\right)}\left(\ln\left(1-\frac{Q_{t}}{\left(100-Q_{0}\right)}\right)\right)$	$T_{d}=e^{-b/a}$

**Table 3:** The parameters determined in the course of release kinetics evaluation of gel formulations F, G, H, where K—release rates for respective model: zero-order kinetics (K_(0)_), 1st-order kinetics (K_(I)_), 2nd-order kinetics (K_(II)_) and the Higuchi model (K_(H)_), β—a shape parameter in the Weibull model, r^2^—correlation coefficient for the regression, SD—standard deviation, MC—methylcellulose, PA1—polyacrylic acid derivative (Carbopol 980 NF), PC-11—modified polyacrylic acid (Aristoflex Velvet).

P:	Mathematical Model Dependent Methods											
	0-Order		1st-Order		2nd-Order		Higuchi			Weibull		BF
	K_(0)_ (%·min^−1^)	SD	K_(I)_ (min^−1^)	SD	K_(II)_ (%^−1·^min^−1^)	SD	K_(H)_ (%·min^−1/2^)		SD	β (–)	SD	
***F:***	**F0 (MC formulation)**											
***C:***	9.0 × 10^−2^	6.59 × 10^−3^	−1.29 × 10^−3^	1.23 × 10^−4^	1.80 × 10^−5^	2.26 × 10^−6^	2.31	1.56 × 10^−1^		6.76 × 10^−1^	3.01 × 10^−2^	H
***r^2^***	0.9638	-	0.9849	-	0.9950	-	0.9980	-		0.9981	-	W
***F:***	**F20 (MC formulation)**											
***C:***	7.59 × 10^−2^	2.45 × 10^−3^	−9.89 × 10^−4^	3.98 × 10^−5^	1.30 × 10^−5^	6.40 × 10^−7^	1.82	5.85 × 10^−2^		5.98 × 10^−1^	2.61 × 10^−2^	II
***r^2^***	0.9798	-	0.9907	-	0.9954	-	0.9920	-		0.9889	-	H
***F:***	**F40 (MC formulation)**											
***C:***	5.70 × 10^−2^	3.33 × 10^−3^	−6.6 × 10^−4^	4.30 × 10^−5^	7.81 × 10^−6^	5.67 × 10^−7^	1.35	7.88 × 10^−2^		7.69 × 10^−1^	9.23 × 10^−2^	II
***r^2^***	0.9899	-	0.9938	-	0.9948	-	0.987	-		0.9947	-	W
***F:***	**G0 (PA1 formulation)**											
***K:***	7.42 × 10^−2^	3.89 × 10^−3^	−9.30 × 10^−4^	5.38 × 10^−5^	1.18 × 10^−5^	7.58 × 10^−7^	1.81	8.94 × 10^−2^		6.47 × 10^−1^	3.75 × 10^−2^	W
***r^2^***	0.9571	-	0.9749	-	0.9878	-	0.9900	-		0.9984	-	H
***F:***	**G20 (PA1 formulation)**											
***R:***	5.9710^−2^	6.52 × 10^−3^	−7.17 × 10^−4^	8.65 × 10^−5^	8.65 × 10^−6^	1.16 × 10^−6^	1.44	1.58 × 10^−1^		6.26 × 10^−1^	5.78 × 10^−2^	W
***r^2^***	0.9708	-	0.9827	-	0.9913	-	0.9900	-		0.9991	-	II
***F:***	**G40 (PA1 formulation)**											
***C:***	4.37 × 10^−2^	5.37 × 10^-3−^	−4.93 × 10^−4^	6.94 × 10^−5^	5.59 × 10^−6^	8.90 × 10^−7^	1.05	1.33 × 10^−1^		6.69 × 10^−1^	6.60 × 10^−2^	W
***r^2^***	0.9730	-	0.9813	-	0.9880	-	0.9940	-		0.9993	-	H
***F:***	**H0 (PC-11 formulation)**											
***C:***	6.72 × 10^−2^	1.73 × 10^−3^	−8.19 × 10^−4^	2.27 × 10^−5^	1.00 × 10^−5^	3.11 × 10^−7^	1.64	4.26 × 10^−2^		6.43 × 10^−1^	2.46 × 10^−2^	W
***r^2^***	0.9522	-	0.9694	-	0.9829	-	0.9930	-		0.9979	-	H
***F:***	**H20 (PC-11 formulation)**											
***C:***	6.29 × 10^−2^	8.43 × 10^−4^	−7.57 × 10^−4^	1.37 × 10^−5^	9.15 × 10^−6^	2.21 × 10^−7^	1.53	2.16 × 10^−2^		6.41 × 10^−1^	2.07 × 10^−2^	W
***r^2^***	0.9594	-	0.9743	-	0.9859	-	0.9920	-		0.9992	-	H
***F:***	**H40 (PC-11 formulation)**											
***C:***	4.83 × 10^−2^	1.31 × 10^−3^	−5.54 × 10^−4^	1.98 × 10^−5^	6.38 × 10^−6^	2.87 × 10^−7^	1.17	3.23 × 10^−2^		6.37 × 10^−1^	3.82 × 10^−2^	W
***r^2^***	0.9665	-	0.9765	-	0.9847	-	0.9930	-		0.9990	-	H
